# Ellipsoid Zone Integrity and Visual Function in Dry Age-Related Macular Degeneration

**DOI:** 10.3390/jpm14050543

**Published:** 2024-05-19

**Authors:** Sari Yordi, Yavuz Cakir, Gagan Kalra, Hasan Cetin, Ming Hu, Joseph Abraham, Jamie Reese, Sunil K. Srivastava, Justis P. Ehlers

**Affiliations:** 1 The Tony and Leona Campane Center for Excellence in Image-Guided Surgery and Advanced Imaging Research, Cleveland Clinic, Cleveland, OH 44195, USA; sfyordi@gmail.com (S.Y.); cakiry@ccf.org (Y.C.); gagan.kalra2796@gmail.com (G.K.); cetinh@ccf.org (H.C.); hum@ccf.org (M.H.); reesej3@ccf.org (J.R.); srivass2@ccf.org (S.K.S.); 2 Cole Eye Institute, Cleveland Clinic, Cleveland, OH 44195, USA; abrahaj7@ccf.org; 3 Department of Quantitative Health Sciences, Cleveland Clinic, Cleveland, OH 44195, USA

**Keywords:** dry age-related macular degeneration, ellipsoid zone, visual acuity

## Abstract

In this longitudinal retrospective image analysis, conducted on patients diagnosed with dry age-related macular degeneration (AMD) and 5 years of follow-up imaging data, the study aimed to investigate the relationship between ellipsoid zone (EZ) integrity on spectral domain optical coherence tomography (SD-OCT) and visual acuity (VA). Using a machine learning-enabled feature extraction tool, quantitative EZ parameters were derived from SD-OCT images. The analysis revealed significant correlations between EZ integrity metrics and VA. Eyes with excellent VA (≥20/25 Snellen) exhibited higher EZ integrity, including less EZ attenuation, thicker ellipsoid zone-retinal pigment epithelium (EZ-RPE) thickness, and higher EZ intensity, in contrast to eyes with worse VA (≤20/40 Snellen). Additionally, eyes with geographic atrophy (GA) in the foveal region displayed compromised EZ integrity compared to those without GA. Notably, baseline EZ integrity metrics were predictive of future VA loss. These findings suggest that quantitative SD-OCT measurements of EZ integrity could potentially detect early changes in dry AMD and serve as valuable indicators for predicting future functional outcomes. Furthermore, these measurements hold promise for use in clinical trial screenings, offering insights into the progression of the disease and its impact on visual acuity. This study underscores the importance of EZ integrity assessment in understanding and managing dry AMD.

## 1. Introduction

Age-related macular degeneration (AMD) is a leading cause of vision loss in people over the age of 50 years [1,2]. The prevalence of AMD is projected to increase globally from 196 million in 2020 to 288 million by 2040 [2]. The traditional classification of AMD split the disease into early and intermediate stages, which are determined primarily by the prevalence and size of drusen in the sub-RPE compartment. Specifically, limited small drusen (less than 63 µm) are classified as early AMD, while multiple medium drusen (between 63 and 125 µm) with associated pigmentary changes are considered to be intermediate AMD [3]. Late-stage AMD is characterized by more severe manifestations of AMD which are divided into dry and wet AMD [4]. Advanced dry AMD describes the disease stage associated with progressive, localized retinal pigment epithelium (RPE) dysfunction, outer retinal attenuation/loss, and associated geographic atrophy (GA), which is a late irreversible manifestation of dry AMD.

Historically, features of dry AMD have been identified with fundus photography and fundus autofluorescence [5,6,7,8]. The Classification of Atrophy Meetings (CAM) working group has demonstrated the potential of using optical coherence tomography (OCT) to diagnose, prognosticate, stage, and monitor dry AMD [9]. The rationale supporting the use of OCT includes widespread availability, high resolution, cross-sectional visualization, and the ability to obtain a more detailed assessment of overall structural disease burden [5]. Previous reports have described GA on spectral domain (SD)-OCT as the total loss of the photoreceptor layer, retinal pigment epithelium (RPE), and the outer neurosensory layer [5,9,10].

The CAM consensus distinguished between the different stages of development of, and the spectrum of, GA, which included incomplete RPE and Outer Retinal Atrophy (iRORA) as a precursor stage and the final complete RPE and Outer Retinal Atrophy (cRORA) or GA [9]. These newer definitions provided more standardized methods of feature identification on OCT, allowing improved consistency in the identification of various stages of the disease and potential application as new endpoints for future treatments of dry AMD. Avacincaptad pegol (IZERVAY™, Astellas, Japan), a complement C5 inhibitor, and Pegcetacoplan (SYFOVRE™, Apellis, MA, USA), a targeted complement C3 inhibitor, represent recent advancements in the treatment of dry AMD. Both drugs have shown significant efficacy in delaying the progression and growth rate of GA lesions, which has led to their recent FDA approvals for managing geographic atrophy secondary to age-related macular degeneration [11,12,13,14,15,16].

The characterization and assessment of dry AMD disease burden with OCT have been greatly enhanced by advanced image analysis, such as using machine learning (ML)-enhanced OCT segmentation and feature extraction. Specific endpoints and biomarkers that are clearly linked to visual function and disease progression are needed, particularly for intermediate AMD, where clinical trial enrichment and the evaluation of therapeutic effect are quite challenging. One specific outer retinal feature which has been strongly associated with functional outcomes is ellipsoid zone (EZ) integrity in multiple retinal disorders, including diabetic macular edema (DME), retinal vein occlusion (RVO), post-operative macular hole (MH), and AMD [17,18,19,20,21,22,23,24,25,26]. The EZ, also referred to as the inner segment/outer segment (IS/OS) junction, is observable in SD-OCT as a reflective layer anterior to the retinal pigment epithelium. Its reflectivity is believed to originate from mitochondria-rich photoreceptor segments in the inner segment ellipsoids, and the loss of reflectivity may indicate pathological states in the outer retina [27].

The relationship between external retinal structures such as EZ and visual acuity has been proven in various retinal pathologies. However, this correlation has not yet been definitively defined in the context of dry AMD. More research needs to be conducted in this area [11,28]. Further analysis and investigation are needed to evaluate the impact of EZ integrity more clearly on visual function, particularly given the rapidly changing therapeutic field in dry AMD. The purpose of this report is to explore the correlation of quantitative EZ integrity parameters in eyes with early to late dry AMD, thereby allowing better characterization of the association between OCT-based anatomic features and functional outcomes.

## 2. Materials and Methods

This was a retrospective, Institutional Review Board (IRB)-approved, longitudinal study evaluating EZ integrity in eyes with dry AMD. This study adhered to the tenets of the Declaration of Helsinki. Due to the retrospective and minimal risk nature of the study, the IRB waived the requirement of informed consent.

Subjects with a diagnostic code for dry age-related macular degeneration, who were followed in our clinic between 2010 and 2019, were evaluated for this analysis. Each case was checked for accurate diagnosis by confirming the documentation of dry AMD or non-neovascular AMD in the clinical notes, and the presence of drusen in the subject’s SD-OCT images at baseline (Year 0). The inclusion criteria comprised dry AMD subjects with 2–5 years macular cube SD-OCT imaging at all timepoints. The exclusion criteria were the presence of wet AMD or any retinal fluid at baseline or within the 5 years of follow-up, a history of anti-VEGF therapy, other concurrent retinal diseases, vitreoretinal surgery, and poor image quality (determined by image graininess, brightness, contrast, and ability to clearly distinguish the retinal layers on SD-OCT). Although not specifically tracked, very few eyes were excluded due to image quality (i.e., <5%).

Demographic and clinical data were collected, which included age, gender, and phakic status. Macular cube data (6 mm × 6 mm, 512 × 128) of the included subjects taken with the Cirrus (Zeiss, Oberkochen, Germany) SD-OCT platform were exported for further analysis.

An ML-enabled feature extraction tool (OCTViewer; Cleveland Clinic) was used to derive quantitative regional and panmacular parameters from OCT macular cubes in an automated manner, as reported previously [20,22,29,30]. In brief, this process involves a trained deep learning model for the automatic analysis of OCT images for the creation of segmentation lines for retinal layers, including the internal limiting membrane (ILM), retinal pigment epithelium (RPE), Bruch’s membrane, and the ellipsoid zone (EZ). These segmentation lines were then reviewed using a layered review process involving a trained expert image analyst with independent consistency by a senior image analyst, and final reconciliation by a retina specialist as needed. Subsequently, compartmental metrics that refer to measurements of specific zones of interest within the retinal tissues, such as EZ-RPE thickness and volumes, were exported. To maximize reading consistency, image reading environments were standardized for location, computer setup, monitor settings, and lighting conditions.

Multiple measures of EZ integrity were evaluated for association with visual function, including mean EZ-RPE central subfield (mean thickness between the EZ and RPE within a 1 mm diameter fovea-centered circle) thickness (CST; µm), mean EZ-RPE central macular (2 mm diameter fovea-centered circle) thickness (CMT; µm), panmacular EZ-RPE volume (compartmental volume between the EZ and RPE in the entire macular cube; mm3), and partial and total EZ attenuation (percentage of macular area covered by EZ-RPE thickness ≤20 µm and 0 µm, respectively). EZ intensity was measured for each a-scan, where the EZ line intensity ranged from 0 to 256 on a grayscale level. If the EZ layer was absent, the EZ intensity was labeled as 0, while maximum brightness (white) was labeled as 256. The EZ intensity value was then averaged for each a-scan across the zone of interest (panmacular, central subfield, central macula). To account for image quality variability, the EZ intensity index (or normalized EZ intensity) was calculated as the [(EZ intensity) × (EZ intensity/RPE intensity)], which standardized the EZ intensity value relative to the RPE layer brightness. The selection of a reference layer for normalization merits careful consideration. In order to substantiate the suitability of the RPE as a reference layer, the participants in this study were categorized into two distinct groups: pseudophakic and phakic. The analysis revealed no statistically significant variation in the EZ integrity metrics (including the EZ-RPE distance, (*p* = 0.34). Furthermore, the EZ intensity normalization methodology was reinforced by demonstrating that the normalized EZ intensity metrics exhibited no statistically significant differentiation between phakic and pseudophakic eyes (*p* = 0.19).

Visual acuity data were obtained from patient charts as Snellen results and were converted to Early Treatment Diabetic Retinopathy Score (ETDRS) letters using logMar conversion for statistical analysis. Comparisons were conducted based on overall visual acuity, including all eyes, eyes with central subfield GA (foveal GA) and eyes without any GA. In addition, analysis for future vision loss was performed to evaluate EZ integrity differences in eyes that lost vision over time compared to eyes that remained stable. For subgroup analyses, patients were divided into two groups based on their Best-Corrected Visual Acuity (BCVA): eyes with excellent VA (≥80 letters or 20/25 Snellen) and those with worse VA (≤70 letters or 20/40 Snellen). Out of 116 eyes, 94 eyes that met these criteria were used for this subgroup analysis.

All statistical analysis was implemented in the statistical software R version 4.2.1. All subjects’ eyes were analyzed as one group before being subdivided into groups according to the presence or absence of foveal GA. For each group, a comparative assessment of mean baseline EZ integrity parameters was conducted between those with excellent VA and those with worse VA. To account for the dependency of two eyes from the same patient, we fitted the linear mixed effects model using the R function “lmer” in the R package “lme4”, where the patient ID was used as the indicator of random effects. Moreover, the Pearson correlation coefficients were also calculated between the visual acuity and EZ integrity parameters and their corresponding *p* values were reported. Longitudinal changes in these parameters were analyzed for eyes with analyzable SD-OCT data five years prior to their most current visit.

## 3. Results

### 3.1. Clinical Characteristics

Initially, the study enrolled 153 patients who received a diagnosis of dry AMD based on International Classification of Diseases (ICD) codes and met the criteria for suitable follow-up periods. Among them, 68 patients were subsequently excluded from the study due to the presence of wet AMD, poor image quality, or subfoveal GA. Consequently, the investigation proceeded with a cohort of one hundred and sixteen eyes of 85 subjects who fulfilled the essential inclusion criteria. Eighty subjects (80/116; 66.7%) were female, and the mean age of all subjects was 78.0 ± 7.7 years. Phakic status was available for all eyes, showing that 56 eyes (56/116; 48.3%) were phakic compared to 60 eyes (60/116; 51.7%) that were pseudophakic. VA was 75.7 ± 8.3 letters (approx. 20/32 Snellen). For the longitudinal assessment, all 116 eyes had analyzable SD-OCT and VA data five years prior (Year 0) and at their latest visit (Year 5). Out of all the eyes in the analysis, thirty-eight eyes (38/116; 32.8%) had GA compared to 78 eyes (78/116; 67.2%) that had no GA in the whole macula. Among the eyes with GA, thirty-two had central subfield involvement (32/38; 84.2%) compared to six (6/38; 15.8%) eyes with peripherally located GA.

### 3.2. Function–Structure Association

Eyes with excellent VA (≥80 letters or 20/25 Snellen) and those with worse VA (≤70 letters or 20/40 Snellen) were compared for EZ integrity features. Fifty-seven eyes (57/116; 49.1%) had excellent VA compared to thirty-seven eyes (37/116; 31.9%) with worse VA. In eyes with foveal GA, 6 eyes (6/32; 18.8%) had excellent VA, whereas 21 eyes (21/32; 65.6%) had worse VA. Of the eyes without GA, forty-nine eyes had excellent VA (49/78; 62.8%), while thirteen eyes had worse VA (13/78; 16.7%). Overall, a significantly greater proportion eyes of with subfoveal GA had worse VA (*p* < 0.001).

**Figure 1:**
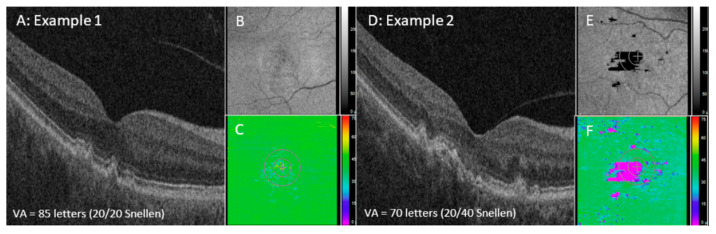
Examples of two eyes (two subjects) with different levels of EZ attenuation and corresponding VA values. Example 1 (**A**–**C**) and Example 2 (**D**–**F**). SD-OCT B-scan of foveal slices from each example (**A**,**D**) with their EZ intensity maps (**B**,**E**) and EZ-RPE thickness maps (**C**,**F**). SD-OCT: spectral domain optical coherence tomography; EZ: ellipsoid zone; RPE: retinal pigment epithelium; BM: Bruch’s membrane; GA: geographic atrophy.

**Figure 2:**
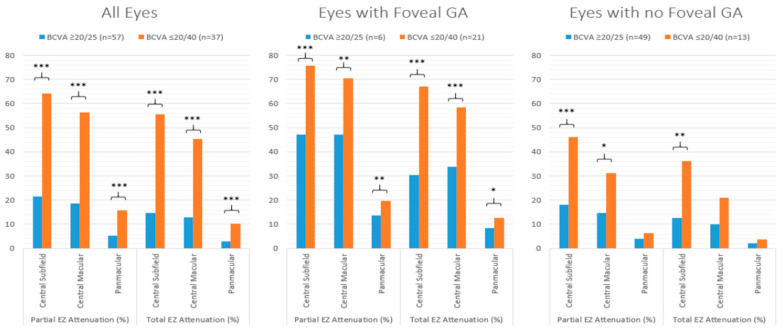
Bar graphs comparing the mean partial and total EZ attenuation in eyes with excellent VA (≥80 letters or 20/25 Snellen) and eyes with worse VA (≤70 letters or 20/40 Snellen), stratified by all eyes, eyes with foveal GA, and eyes with no GA. *** *p* value ≤ 0.001; ** *p* value ≤ 0.01; * *p* value ≤ 0.05. EZ: ellipsoid zone; VA: visual acuity; GA: geographic atrophy.

**Figure 3:**
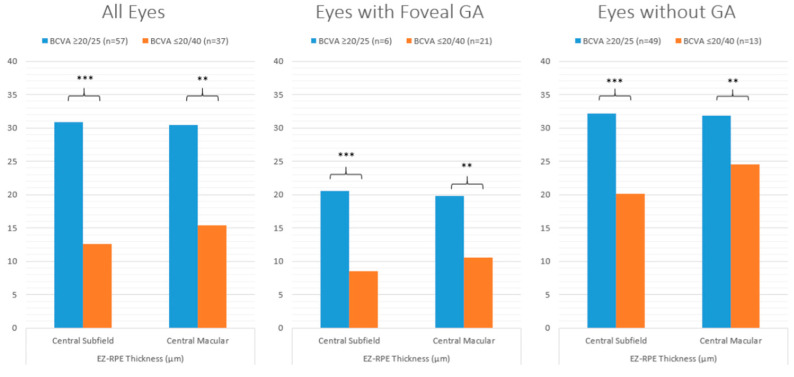
Bar graphs comparing the mean central subfield/macular EZ-RPE thickness in eyes with excellent VA (≥80 letters or 20/25 Snellen) and eyes with worse VA (≤70 letters or 20/40 Snellen) stratified by all eyes, eyes with foveal GA, and eyes with no GA. *** *p* value ≤ 0.001; ** *p* value ≤ 0.01; EZ: ellipsoid zone; RPE: retinal pigment epithelium; VA: visual acuity; GA: geographic atrophy.

**Figure 4:**
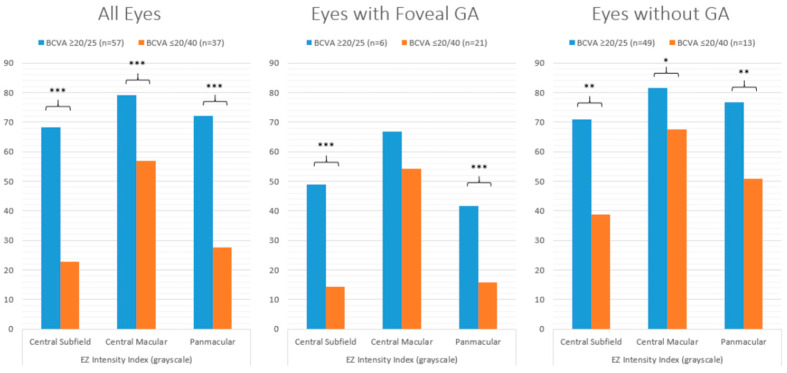
Bar graphs comparing the mean EZ intensity index in eyes with excellent VA (≥80 letters or 20/25 Snellen) and eyes with worse VA (≤70 letters or 20/40 Snellen), stratified by all eyes, eyes with foveal GA, and eyes with no GA. *** *p* value ≤ 0.001; ** *p* value ≤ 0.01; * *p* value ≤ 0.05. EZ: ellipsoid zone; VA: visual acuity; GA: geographic atrophy.

Representative examples of two eyes (two subjects) with varying EZ attenuation and corresponding VA are shown in Figure 1. For all eyes, those with excellent VA demonstrated significantly less partial and total EZ attenuation in all regions (central subfield, *p* ≤ 0.001; central macula, *p* ≤ 0.001; panmacular *p* ≤ 0.001), as well as increased mean EZ-RPE thickness in the central subfield (*p* ≤ 0.001), central macula (*p* ≤ 0.001), and fovea (*p* ≤ 0.001) (Figure 2 and Figure 3). Eyes with excellent VA also showed significantly increased panmacular EZ-RPE volume (*p* ≤ 0.001). Moreover, the EZ intensity index was significantly higher in eyes with excellent VA compared to those with worse VA (*p* ≤ 0.001) (Figure 4).

Similar findings were identified in eyes with foveal GA. Specifically, eyes with excellent VA had significantly less partial and total attenuation, higher EZ-RPE thickness and volume, and a higher panmacular and central subfield EZ intensity index. Among eyes without GA, those with excellent VA demonstrated significantly less partial and total EZ attenuation, higher EZ-RPE thickness, and a higher EZ intensity index within the central subfield and central macular subfield. However, panmacular EZ attenuation values were similar between eyes with excellent VA and those with worse VA, and panmacular EZ-RPE volume was also equivocal. These findings are outlined in more detail in Table 1.

When correlating EZ integrity metrics and VA for all eyes, all assessments demonstrated significant correlations. The Pearson correlation coefficients (R) of total and partial EZ attenuation ranged from −0.43 to −0.53, indicating medium-to-strong negative associations. EZ-RPE CST and panmacular volume measurements showed significant medium positive associations, with correlation coefficients ranging from +0.47 to +0.55. The EZ intensity index in the central subfield, central macula, and whole macula was also significantly correlated with VA (r = +0.52, +0.47, and +0.54, respectively; *p* ≤ 0.001).

In eyes with foveal GA, central subfield and central macular partial attenuation and total attenuation were significantly correlated with GA, with correlation coefficients ranging from −0.45 to −0.54. Mean central subfield and central macular EZ-RPE thickness values were also significantly correlated (r = +0.46 and 0.52, respectively). However, panmacular partial and total attenuation, as well as panmacular EZ-RPE volume, were not significantly correlated with visual acuity. Conversely, EZ intensity indices were significantly correlated with VA in the central subfield, central macula, and panmacular (r = 0.49, 0.37, and 0.52, respectively; *p* ≤ 0.05) regions. In eyes without GA, partial EZ attenuation was only significantly associated with VA in the central subfield (r = −0.28), whereas other regions were not correlated. Total EZ attenuation and EZ-RPE volume were not correlated with VA in any region. However, EZ intensity indices were once again significantly correlated with VA in all regions (r = +0.30, +0.27, +0.26, respectively; *p* ≤ 0.05). The full list of correlation coefficients (r) is displayed in Table 2.

### 3.3. Baseline EZ Integrity and Future Vision Loss

**Figure 5:**
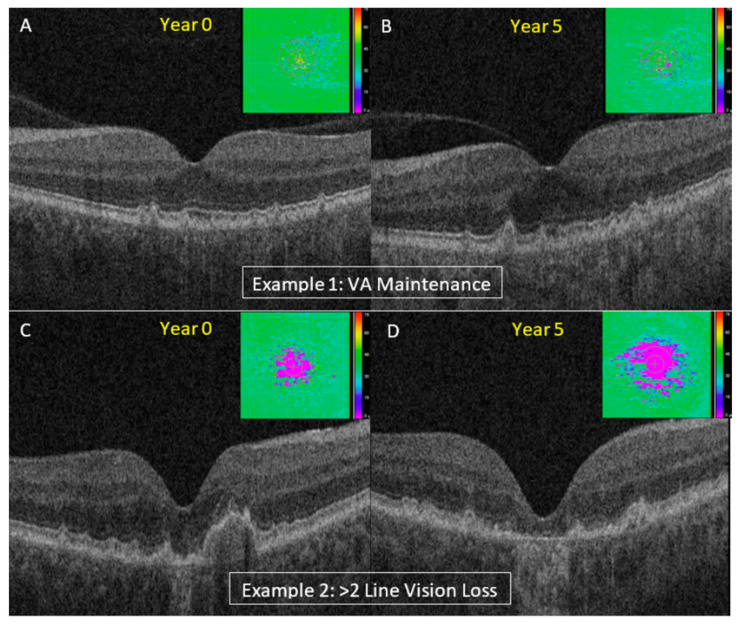
Examples of two subjects (two eyes) with varying EZ attenuation at Year 0 showing different progression of VA during 5-year follow-up. Example 1 shows minimal EZ attenuation and excellent VA at Year 0 (85 letters or 20/20 Snellen) (**A**) with subsequent maintenance of excellent VA at Year 5 (80 letters or 20/25 Snellen) (**B**). Example 2 shows more significant EZ attenuation and worse VA at Year 0 (70 letters or 20/40 Snellen) (**C**) with >2-line worsening of VA by Year 5 (58 letters of 20/80 Snellen) (**D**). EZ-RPE thickness maps on top right of (**A**–**D**) indicate areas of EZ attenuation. SD-OCT: spectral domain optical coherence tomography; EZ: ellipsoid zone.

Eyes that worsened substantially in VA over time (i.e., a loss of two lines or more) also showed significant differences in EZ integrity parameters at baseline compared to those with less VA worsening/improvement. A representative example is shown in Figure 5. There were 25 eyes (25/116; 21.2%) that worsened by at least two lines (“≥2-line group”) compared to 79 eyes (79/116; 68.1%) that experienced less than one-line worsening (“≤1-line group”) between Year 0 and Year 5. At Year 0, partial and total EZ attenuation in all regions (central subfield, central macular, panmacular) were significantly higher in eyes that worsened by two lines between Year 0 and Year 5 (*p* ≤ 0.001, all). EZ-RPE thickness in central subfield and central macula was significantly lower in the “≥2-line group” (*p* ≤ 0.001, both) as well as the EZ-RPE panmacular volume (*p* ≤ 0.001). Moreover, the EZ intensity index in all regions was significantly worse in the “≥2-line group” compared to the “≤1-line group” in the central subfield, central macular, and panmacular regions (*p* ≤ 0.001, all) (Table 3).

These differences also held true when controlling for excellent VA at baseline. Eyes that had excellent VA at Year 0 (VA ≥ 20/25) and maintained excellent VA at Year 5 (“stable” group) were compared to eyes that started with excellent VA at Year 0 and worsened to 20/40 VA or less at Year 5 (“worsened” group). There were 80 eyes with excellent VA at Year 0. Of those, 16 “worsened” (16/80; 20.0%) compared to 50 eyes in the “stable” group (50/80; 62.5%). At Year 0, eyes that were in the “worsened group” had significantly greater partial and total EZ attenuation in all regions (*p* ≤ 0.001, all), decreased EZ-RPE thickness (*p* ≤ 0.001), decreased EZ-RPE volume (*p* ≤ 0.001), and decreased central subfield, central macular, and panmacular EZ intensity indices (*p* ≤ 0.001, all) (Table 4).

## 4. Discussion

In this longitudinal 5-year image analysis study, VA in dry AMD was strongly linked to multiple EZ integrity parameters regardless of GA status. In addition, baseline EZ integrity parameters were also significantly associated with subsequent vision loss over time, even in eyes with excellent baseline VA. This suggests that quantitative OCT-based EZ integrity measures may be an objective biomarker that can show early changes in patients with dry AMD preceding a decline in VA and is overall clearly linked to function.

The EZ is comprised mainly of mitochondria within the ellipsoid layer of the outer portion of the inner segments of the photoreceptors. Disruption or absence of the EZ has been studied extensively and has been ascribed to a variety of retinal conditions, including cone dystrophy [31], achromatopia [32], and age-related macular degeneration (AMD) [27,33,34,35,36,37,38]. Absence or disruption of this layer has been shown to correlate with visual outcomes and disease severity [34]. In one study, Pilotto and colleagues reported that retinal areas with disruption of EZ have a higher risk of progression to extensive scotoma in eyes with geographic atrophy (GA) secondary to AMD than areas with intact EZ [39].

In the current study, a significant correlation was observed between baseline percentage area of partial and complete EZ attenuation, and 5-year VA values, which is consistent with previous studies [17,18,20]. Further, the current analysis also showed a significant inverse correlation between the baseline central EZ-RPE thickness and the 5-year VA. These results could enable the use of partial/total EZ attenuation and EZ-RPE thickness as objective indications of disease severity and potentially predict future functional outcomes in patients with dry AMD.

Additionally, the current study showed a significant correlation between the EZ intensity index and VA. EZ intensity (i.e., EZ reflectivity brightness) is another quantitative OCT-based feature that has been examined as a potential biomarker of risk for the progression of dry AMD. It is believed that photoreceptor mitochondria and their organization are responsible for producing the EZ signal on OCT, and its reflectivity is thought to be a reflection of the metabolic activity of photoreceptor mitochondria [17,40,41,42]. Therefore, the EZ signal may serve as a unique surrogate biomarker of the activity and health of photoreceptors [33,34,35,36]. The correlation of EZ intensity with VA places emphasis on the future exploration of this biomarker as a potential endpoint for clinical trials, although there are potentially greater challenges in utilizing this measure compared to the EZ-RPE thickness measures based on image variability.

Promising results have been shown in recent clinical trials which have targeted various elements in the pathophysiology of dry AMD, such as the complement pathway and mitochondrial oxidation, and employed anti-inflammatory and cell-based therapies [43,44]. Although GA is irreversible, these novel therapies have demonstrated the potential to halt or slow the progression of GA [43,44]. There are numerous promising ongoing trials clinical trials that can benefit from objective and quantitative OCT-based disease monitoring [44,45]. A recently FDA-approved therapy for GA, pegcetacoplan (SYFOVRE™, Apellis), specifically inhibits complement C3 and has demonstrated delays in the advancement of dry AMD and decelerating the growth of GA lesions [11,13,14]. Riedl and colleagues have also demonstrated the benefit of pegcetacoplan in slowing down photoreceptor layer loss in eyes with dry AMD via quantitative machine learning-enhanced analysis [46]. In addition, elamipretide (Stealth Biotherapeutics, MA, USA) in a prespecified analysis in the ReCLAIM-2 Phase 2 trial demonstrated a significant reduction in the progressive loss of EZ integrity in dry AMD [27]. In GATHER1/2, avacincaptad pegol (Iveric BIO, NJ, USA), a C5 inhibitor, has also demonstrated reduction in photoreceptor loss compared to sham as measured by EZ attenuation (presented at ASRS conference 2023, Seattle, WA, USA).

The strengths of the current report include the relatively long follow-up period of up to 5 years, natural history setup, and quantitative objective measurement of EZ parameters on SD-OCT. In addition, the use of a robust previously validated deep learning-enabled system for OCT segmentation made it possible to minimize segmentation corrections and improve consistency.

This study has important limitations to consider. The lack of protocol refraction visual acuity measurements and the relatively small size of our dataset also contribute to the study’s limitations. As a result, more independent studies are needed to determine generalizability to a larger population. In addition, eyes with intermediate AMD often exhibit a diverse composition of both traditional drusen and pseudodrusen. Acknowledging this complexity, pseudodrusen were not excluded, and EZ segmentation was performed based on their overall appearance in regions similar to the segmentation method applied to traditional drusen areas in this study. This analysis also focused on the univariate analysis of EZ integrity features with visual function. Additional validation with multivariate assessments was not performed and is planned for future larger validation studies.

The significant associations between quantitative EZ integrity parameters on SD-OCT with both visual function and future vision loss provide a critical opportunity to potentially risk-stratify eyes with dry AMD and enrich clinical trials for eyes at the greatest risk for functional change. This also presents a critical structure–function relationship that supports EZ integrity and photoreceptor preservation as an appropriate end point for future therapeutics. Finally, the use of EZ integrity as an imaging biomarker may provide critical support to clinicians to identify those patients who may be ideal candidates for therapeutic intervention. Future research will include both prospective and large-scale retrospective validation of these findings.

## Figures and Tables

**Table 1:** Comparison between mean values of subjects with 20/25 or better on Snellen chart (≥20/25) and subjects with 20/40 BCVA or worse (≤20/40) separated by region. *p* values were calculated from the difference between the means of EZ parameters in eyes that had good vision (BCVA ≥ 20/25) compared to those with worse vision (BCVA ≤ 20/40). Bolded values indicate a statistically significant difference. *** *p* value ≤ 0.001; ** *p* value ≤ 0.01; * *p* value ≤ 0.05.

		All Eyes			Eyes with Foveal GA			Eyes with No GA		
Parameter	Region	BCVA ≥20/25 (n = 57)	BCVA ≤20/40 (n = 37)	*p* Value	BCVA ≥20/25 (n = 6)	BCVA ≤20/40 (n = 21)	*p* Value	BCVA ≥20/25 (n = 49)	BCVA ≤20/40 (n = 13)	*p* Value
Partial EZ attenuation (%)	Central subfield	**21.5**	**64.2**	***	**47.1**	**75.6**	***	**18.1**	**46.2**	***
	Central macular	**18.5**	**56.4**	***	**47.2**	**70.5**	**	**14.8**	**31.2**	*
	Panmacular	**5.4**	**15.7**	***	**13.7**	**19.6**	**	4.0	6.2	ns
Total EZ attenuation (%)	Central subfield	**14.6**	**55.6**	***	**30.5**	**67.0**	***	**12.6**	**36.2**	**
	Central macular	**12.8**	**45.2**	***	**33.7**	**58.3**	***	10.1	21.0	ns
	Panmacular	**3.0**	**10.2**	***	**8.3**	**12.6**	*	2.2	3.6	ns
EZ-RPE thickness (µm)	Central subfield	**30.9**	**12.6**	***	**20.6**	**8.5**	***	**32.2**	**20.1**	***
	Central macular	**30.4**	**15.4**	***	**19.8**	**10.6**	**	**31.8**	**24.5**	**
EZ-RPE volume (mm^3^)	Panmacular	**1.238**	**1.084**	***	**1.151**	**1.029**	**	1.250	1.213	ns
EZ intensity index (grayscale)	Central subfield	**68.2**	**22.7**	***	**48.9**	**14.4**	***	**70.9**	**38.8**	**
	Central macular	**79.1**	**56.9**	***	66.9	54.3	ns	**81.5**	**67.6**	*
	Panmacular	**72.2**	**27.7**	***	**41.6**	**15.7**	***	**76.6**	**50.8**	**

**Table 2:** Pearson correlation (r) values for all EZ metrics separated by region. All values were significant (*p* < 0.001). Color scale signifies strength of association between BCVA and respective SD-OCT metric. Green = positive correlation; red = negative correlation. Bold values are significant (*p* ≤ 0.05).

Parameter	Region	All Eyes	Eyes with Foveal GA	Eyes with No GA
Partial EZ attenuation (%)	Central subfield	**−0.50**	**−0.45**	**−0.28**
	Central macular	**−0.53**	**−0.49**	−0.21
	Panmacular	**−0.43**	−0.30	−0.14
Total EZ attenuation (%)	Central subfield	**−0.50**	**−0.49**	−0.24
	Mid-subfield	**−0.52**	**−0.54**	−0.15
	Panmacular	**−0.46**	−0.34	−0.12
EZ-RPE thickness (µm)	Central subfield	**0.53**	**0.46**	**0.30**
	Central macular	**0.55**	**0.52**	**0.26**
EZ-RPE volume (mm^3^)	Panmacular	**0.42**	0.34	0.12
EZ intensity index (grayscale)	Central subfield	**0.52**	**0.49**	**0.30**
	Central macular	**0.47**	**0.37**	**0.27**
	Panmacular	**0.54**	**0.53**	**0.26**
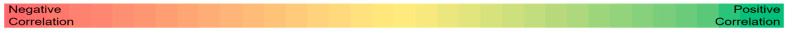				

**Table 3:** Comparison of Year 0 mean EZ integrity parameter values between eyes that worsened by at least 2 lines (“≥2-line group”) and eyes that experienced less than 1 line worsening in VA (“≤1-line group”) between Year 0 and Year 5. *p* values compare the mean value between “worse” and “stable/improved” cohorts.

Year 0 Parameter	Region	≥2-Line Group (n = 25)	≤1-Line Group (n = 79)	*p* Value
Partial EZ attenuation (%)	Central subfield	39.9	10.7	≤0.001
	Central macular	31.6	7.8	≤0.001
	Panmacular	5.5	1.9	≤0.001
Total EZ attenuation (%)	Central subfield	27.5	5.7	≤0.001
	Central macular	20.9	4.0	≤0.001
	Panmacular	3.1	0.8	≤0.001
EZ-RPE thickness (µm)	Central subfield	22.8	34.5	≤0.001
	Central macular	25.2	34.0	≤0.001
EZ-RPE volume (mm^3^)	Panmacular	1.228	1.294	≤0.01
EZ intensity index (grayscale)	Central subfield	55.5	81.8	≤0.001
	Central macular	76.2	88.4	≤0.05
	Panmacular	61.8	88.0	≤0.001

**Table 4:** Year 0 EZ integrity parameters of eyes that had excellent visual acuity (VA ≥ 20/25) at Year 0 that either worsen to at least 20/40 (“worsened” group) or maintain VA at 20/25 or better (“stable/improved” group) by Year 5. *p* values compare the mean value between “worsened” and “stable/improved” groups. Bold *p* values are significant.

Year 0 Parameter	Region	Worsened (n = 16)	Stable/Improved (n = 50)	*p* Value
Partial EZ attenuation (%)	Central subfield	33.6	5.6	≤0.001
	Central macular	27.4	4.8	≤0.001
	Panmacular	5.1	1.5	≤0.001
Total EZ attenuation (%)	Central subfield	20.7	2.3	≤0.001
	Central macular	16.4	2.2	≤0.001
	Panmacular	2.6	0.6	≤0.001
EZ-RPE thickness (µm)	Central subfield	26.1	36.5	≤0.001
	Central macular	27.1	35.0	≤0.001
EZ-RPE volume (mm^3^)	Panmacular	1.240	1.294	≤0.05
EZ intensity index (grayscale)	Central subfield	63.8	88.2	≤0.001
	Central macular	76.7	91.5	≤0.05
	Panmacular	67.5	93.1	≤0.001

## Data Availability

Data are contained within the article.
